# Making the “Right” Health Care Decisions: Why Values Matter

**DOI:** 10.1371/journal.pmed.1000136

**Published:** 2009-08-25

**Authors:** The PLoS Medicine Editors

Shared decision making in health care can be defined as the process of “…defining problems, presenting options, and providing high-quality information so patients can participate more actively in care…” [1]. This model of decision making is rooted in several core principles of medical ethics, but perhaps most strongly that of patient autonomy [2]. Autonomy—the right to self-determination—entails a process of informed and meaningful consent to the care a patient is to receive [3]. The idea of informed consent clearly goes beyond a simple procedure of form-filling, and requires that the nature of an intervention, the potential alternatives, the likely risks and benefits, and the implications are clearly laid out and mutually understood before a patient and clinician can agree on the course of action to be undertaken. In a Policy Forum published in *PLoS Medicine*, Michael Wilkes and Margaret Johns set out four key characteristics of the types of decisions that best lend themselves to shared decision making [4]: those where “effectiveness of the outcome is uncertain; …where the risks and benefits are sizeable or nearly equal; …where the patient is able and willing to participate; …[and] where the patient can understand the trade-offs.” An obvious requirement for the fourth characteristic—the understanding of trade-offs—is that a patient interprets data regarding risk and can integrate that data into their own system of values—an issue we discuss within this Editorial.

Indeed, very many common decisions would fit Wilkes and Johns' criteria for shared decision making. In such contexts, decision aids may help patients weigh up the factors that bear upon different treatment options. For example, a randomized trial [5] evaluated the effects of a visual “decision board,” presenting the available treatment options, adverse effects, and effects of different treatments on survival and quality of life for women with early-stage breast cancer. In the trial, the board increased women's knowledge of treatment options, and reduced “decisional conflict” (or personal uncertainty in the decision). Similarly, systematic reviews of trials evaluating decision aids in general have concluded that such tools “…do a better job than usual care interventions in improving people's knowledge regarding options, reducing their decisional conflict related to feeling uninformed and unclear about personal values, decreasing the proportion of people remaining undecided, and stimulating people to take a more active role…” [6]. Decision aids such as those described therefore seem to provide a valuable route towards the desired goal of more fully informed consent, and a shared decision making process.

A key challenge, however, to the premise of shared decision making is the observation that a patient's choice of their preferred treatment will change *depending on the way that key data are presented*. For example, survival data can be represented in a “positive frame”—chance of survival—or a “negative frame”—chance of dying. A patient's choice regarding treatment options will change, depending on which type of presentation is given, even if the actual data are equivalent [7]. However, little research has been done to explore the dependencies between the way that key statistics are presented, and a patient's choice in relation to their own prior values.

In this issue of *PLoS Medicine*, we publish two papers reporting results of Internet-based randomized trials that investigate which types of presentation help people to make decisions most consistent with their own values [8],[9]. In one study [8], a trial randomizing 2,978 participants to view six alternative presentations of the likely reduction in risk of coronary heart disease when taking statins, Cheryl Carling and colleagues report that some ways of presenting quantitative data—for example, framing outcomes in terms of relative risk reduction—resulted in higher numbers of participants indicating that they would choose to take the preventive intervention. This effect held irrespective of a participant's prior values. For example, participants who did not place high importance on the prevention of coronary heart disease could still be “persuaded” to take statins when they were given data in the form of a relative risk reduction statistic. However, participants who were less concerned about potentially having coronary heart disease were still less likely to choose statins. The relationship between a participant's values and their decision to take statins was found to be similar for all ways of presenting risk evaluated in this study. Therefore, Carling and colleagues suggest that natural frequencies are the most appropriate tool to use in presenting this type of data, given that participants reported these as easiest to understand and that they gave participants the most confidence in their decision.

The researchers also conducted a separate trial evaluating participants' decisions as to whether to visit the doctor for an antibiotic prescription for sore throat [9]. In this trial, 1,760 people saw four different graphical displays representing the effects of antibiotics on the symptoms of sore throat, or no information. The results of the trial suggest that bar graphs, showing the likely duration of symptoms, helped participants make the decisions most consistent with their values, and were most often preferred. Both trials show that as a participant's prior values change, their decision will also change.

It is clear from these studies, and from systematic reviews of “information framing” that there is the potential for shared decision making to be biased through the adoption of more persuasive presentations—such as relative statistics. As a result, the underlying principle of shared decision making—that of empowering patients to make decisions most compatible with their values—can be undermined. However, the two trials published in this issue of *PLoS Medicine* do suggest that certain ways of framing information—such as the use of natural frequencies—can be adopted that are both readily understandable by participants and consistent with their values. Moreover, these studies illustrate how difficult it can be to generate reliable evidence on the ways in which people make real-life decisions: both trials found recruitment difficult, and both explore hypothetical scenarios rather than actual decision making in a health care context by patients. In real life, the decisions that need to be made are perhaps not as straightforward as those evaluated by Carling and colleagues. Solid evidence on likely outcomes of different treatment options may not exist, and even high-quality quantitative evidence is but one factor within the emotional, social, and cultural context of shared decision making [1],[10]. Trials such as those discussed here may provide evidence regarding the most appropriate method for presenting data in an unbiased way to patients. But in order for shared decision making to support patient autonomy, health care providers must recognize the role of their own values and understand and respect those of the patient, in the decision that is ultimately made.

## References

[1] Epstein RM, Peters E (2009). Beyond information: Exploring patients' preferences.. JAMA.

[2] Gillon R (1994). Medical ethics: Four principles plus attention to scope.. BMJ.

[3] Meisel A, Kuczewski M (1996). Legal and ethical myths about informed consent.. Arch Intern Med.

[4] Wilkes M, Johns M (2008). Informed consent and shared decision-making: A requirement to disclose to patients off-label prescriptions.. PLoS Med.

[5] Whelan T, Levine M, Willan A, Gafni A, Sanders K (2004). Effect of a decision aid on knowledge and treatment decision making for breast cancer surgery: A randomized trial.. JAMA.

[6] O'Connor AM, Bennett CL, Stacey D, Barry M, Col NF (2009). Decision aids for people facing health treatment or screening decisions.. Cochrane Database Syst Rev.

[7] Moxey A, O'Connell D, McGettigan P, Henry D (2003). Describing treatment effects to patients: How they are expressed makes a difference.. J Gen Intern Med.

[8] Carling CLL, Kristoffersen DT, Montori VM, Herrin J, Schünemann HJ (2009). The effect of alternative summary statistics for communicating risk reduction on decisions about taking statins: A randomized trial.. PLoS Med.

[9] Carling CLL, Kristoffersen DT, Flottorp S, Fretheim A, Oxman AD (2009). The effect of alternative graphical displays used to present the benefits of antibiotics for sore throat on decisions about whether to seek treatment: A randomized trial.. PLoS Med.

[10] Kravitz RL, Melnikow J (2001). Engaging patients in medical decision making.. BMJ.

